# A Study of Thermistor Performance within a Textile Structure

**DOI:** 10.3390/s17081804

**Published:** 2017-08-05

**Authors:** Theodore Hughes-Riley, Pasindu Lugoda, Tilak Dias, Christophe L. Trabi, Robert H. Morris

**Affiliations:** 1 Advanced Textiles Research Group, School of Art & Design, Nottingham Trent University, Bonington Building, Dryden Street, Nottingham NG1 4GG, UK; pasindu.lugoda2013@my.ntu.ac.uk (P.L.); tilak.dias@ntu.ac.uk (T.D.); 2 School of Science and Technology, Nottingham Trent University, Clifton Lane, Nottingham NG11 8NS, UK; christophe.trabi@ntu.ac.uk (C.L.T.); rob.morris@ntu.ac.uk (R.H.M.)

**Keywords:** wearable electronics, smart textiles, temperature-sensing, diabetic ulcers, thermistor, wound management, sensor network

## Abstract

Textiles provide an ideal structure for embedding sensors for medical devices. Skin temperature measurement is one area in which a sensor textile could be particularly beneficial; pathological skin is normally very sensitive, making the comfort of anything placed on that skin paramount. Skin temperature is an important parameter to measure for a number of medical applications, including for the early detection of diabetic foot ulcer formation. To this end an electronic temperature-sensor yarn was developed by embedding a commercially available thermistor chip into the fibres of a yarn, which can be used to produce a textile or a garment. As part of this process a resin was used to encapsulate the thermistor. This protects the thermistor from mechanical and chemical stresses, and also allows the sensing yarn to be washed. Building off preliminary work, the behaviour and performance of an encapsulated thermistor has been characterised to determine the effect of encapsulation on the step response time and absolute temperature measurements. Over the temperature range of interest only a minimal effect was observed, with step response times varying between 0.01–0.35 s. A general solution is presented for the heat transfer coefficient compared to size of the micro-pod formed by the encapsulation of the thermistor. Finally, a prototype temperature-sensing sock was produced using a network of sensing yarns as a demonstrator of a system that could warn of impending ulcer formation in diabetic patients.

## 1. Introduction

The aim of this publication is to report the new knowledge that has been established in order to better understand how the performance of a thermistor is affected when embedded within the fibers of a yarn with a focus on the polymer micro-pod crafted to protect the thermistor from the mechanical and chemical stresses that a yarn would encounter when contained in a garment.

Prototype temperature-sensing yarns had their response to temperature changes characterized before being incorporated into a prototype temperature-sensing sock, showing a potential future use of temperature-sensor yarns. The motivation for developing temperature-sensor yarns by using commercially available thermistors is summarized below.

Textiles can provide a platform for embedding sensors when creating wearable medical devices. These are the perfect substrate for many applications as textiles are conformable, drapeable, flexible, and breathable making them comfortable to wear. Textiles can be constructed from individual yarns made of fibres, and formed into a structure using methods such as knitting or weaving.

Historically mercury-in-glass thermometers were used to monitor temperature in a clinical setting, however due to the safety issues relating to mercury these have been relegated to laboratory use only. Existing skin temperature measurements are performed using either thermistors or thermocouples, which are both accurate and inexpensive [1]. Clinical thermistors of a very high accuracy (±0.01 °C) have previously been reported [2]. Resistance temperature detectors can also be used in a similar way. Beyond this, thermocouples have been employed for skin temperature measurements, as have resistance wires and thermopiles [3]. Thin-film sensors have also gained interest in recent years as a method to monitor skin temperature [4]. Liquid crystal thermometers are also sometimes employed to take skin temperature measurements in a domestic setting. As well as contact measurements, non-contact skin measurements can be obtained using radiometric methods. In recent years infra-red (IR) thermometry has become a popular alternative method with the performance of infrared thermometers seen to be equal or superior to thermistors for skin-surface thermometry [5,6] based on the pioneering work by Hardy [7].

In this study, yarn encapsulation technology was used to embed small thermistors discretely into yarns, and then textiles [8,9], to create sensing garments capable of spatially resolved temperature measurements. Textiles are an ideal medium for employing temperature-sensing on the body, as temperature changes in the skin can indicate underlying pathologies [10,11,12,13,14,15,16,17]. This requires direct skin contact for the encapsulated sensor and therefore comfort is essential. Further, traditional thermometry techniques are not well suited to ambulatory temperature measurement or indeed regular contact measurements of a single point on the skin as misplacement of the temperature-sensing element between measurements could result in erroneous conclusions. This is also true for IR measurements, where the IR head may not be aimed at the correct location. Misplacement is less likely with a device that can be worn and does not need to be removed. A tight-fitting garment such as a sock is not likely to move significantly during use, making misplacement errors less likely.

The proposed system is superior to existing textile-based temperature-sensors as these are incapable of localised measurements. These systems typically use the voltage drop of a metal conducting wire inlayed within a knitted structure to measure temperature [18]. Other fibre-based systems proposed have seen a Polyvinylidene fluoride (PDVF) monofilament coated in a thermo-sensitive layer to create temperature-sensing fibres that can be integrated into garments. This allowed for a temperature measurement across single fibres of 2 to 6 cm lengths but not for point measurements [19]. Flexible, wearable temperature sensors not embedded within textiles are significantly less comfortable to wear as these do not exhibit textile characteristics, such as the ability to bend and shear [4,20,21]. The concept discussed in this work is similar to the e-fibre temperature sensor technology proposed by Cherenack et al., with a major difference being that the e-yarns in this work use a fibrous sheath to give the yarns a true textile feel [22]. This study builds on previous preliminary work [8] where a temperature-sensing yarn was proposed. Here, the temperature-sensing yarn design has been fully characterised and a garment (a sock) capable of spatially resolved temperature measurements is presented.

An area of particular interest in the development of this technology is to warn of foot ulceration in diabetics. It is known that there is a localized skin temperature change in the feet prior to ulcer formation in diabetic high-risk patients [11,12,14,17], presenting as a >2 °C change. Therefore, a temperature-sensing sock able to detect temperature within an accuracy of ±0.5 °C would offer a useful solution by indicating that an ulcer may be forming. An extensive study (173 diabetes patients with a history of ulceration over a 15-month period) performed by Lavery et al. had patients measure six points on the foot—the big toe, the first, third, and fifth metatarsal heads, the heel, and the midfoot. Patients with an at-home device to monitor skin temperature at these points of the foot were less likely to develop ulcers (with only 8.5% of the study group developing ulcers) than other groups (with around 30% of the study group developing ulcers) helping validate the concept [12]. It is likely that these locations were chosen by Lavery et al. as diabetic foot ulcers are commonly known to most often form on the base of the big toe, metatarsal heads, and heel. As the relative temperature will only change in locations where an ulcer is about to form, in the first instance a temperature-sensing sock should measure at these three locations.

Another application of the technology would be to create a temperature-sensing wound dressing, as wound infection can also be identified by a localised change in temperature [13,15,16] however the current literature does not provide guidance of the actual temperature variations that relate to wound infection.

**Figure 1:**
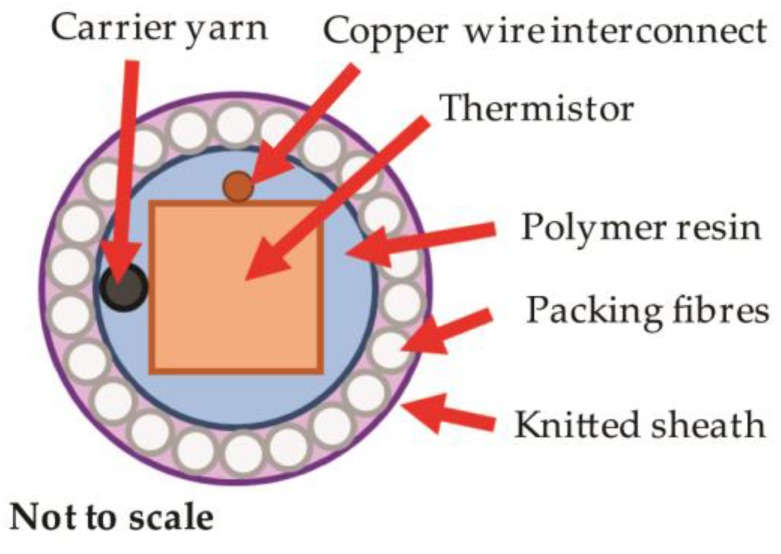
A cross-sectional schematic of the proposed encapsulation for a thermistor within a yarn. There are three layers—the polymer resin, packing fibres, and a knitted sheath. Each will retard the flow of heat to the thermistor, affecting the sensor’s response. The yarn would also include a carrier yarn within the resin encapsulation to improve the tensile strength of the final yarn.

In order to successfully create temperature-sensing yarns and textiles that can provide correct temperature measurement, it is vital to understand the effects of heat transfer created by the inclusion of encapsulation layers around the thermistor within the yarn. The encapsulation process is essential in order to protect the thermistor from the mechanical and chemical stresses that a yarn would encounter in use. The standard encapsulation process uses three layers; a polymer resin, packing fibres, and a knitted sheath as shown in Figure 1.

The packing fibres and knitted sheath should have a minimal effect on heat transfer to and from the thermistor, given the relatively low mass of the materials and their porous structure. It was the polymer micro-pod that would retard heat flow more significantly. This potentially alters the step response time of the sensor (the time taken for the sensor to provide a representative measurement after a change [23]) or the absolute temperature measurement.

## 2. Materials and Methods

### 2.1. Electronic Temperature-Sensing Yarn Construction

The electronic temperature-sensing yarn construction followed the general production steps described elsewhere in the literature [8,9]. The electronic temperature-sensing yarns were constructed using a single eight strand copper wire (outer diameter = 140 μm; 16 Ωm^−1^ copper alloy; Red House Global, Colmworth, UK) soldered as interconnects to a Murata 10 kΩ, 100 mW 0402 surface-mount device (SMD) Negative Temperature Coefficient (NTC) thermistor (NCP15XH103F03RC; Murata, Kyoto, Japan), which was chosen as it was small enough to embed within a thin yarn (0.5 mm × 0.5 mm × 1 mm) and was sensitive to the temperature range of interest (approximately 25–38 °C) [24]. A thermoplastic monofilament yarn spun from liquid crystal polymer (Vectran™, Kuraray America Inc., Houston, TX, USA) was used as a carrier fibre to provide mechanical strength to the fine copper wire. The thermistor, carrier and interconnects were encapsulated in a cylindrical polymer micro-pod using 5 μL of UV curable resin (Multi-Cure^®^ 9001-E-V-3.5, Dymax, Torrington, CT, USA; thermal conductivity: 0.2 Wm^−1^·K^−1^).

In order to conduct studies to understand the effects of the micro-pod on the sensor’s response, a number of different thicknesses of micro-pod were investigated with diameters of 0.70 mm, 1.01 mm, 1.50 mm, 1.76 mm, and 1.90 mm. Tests were also conducted using un-encapsulated thermistors. To test the effects of the micro-pod, these yarn samples were not completed with outer fibrous layers in order to fully understand the effects of the resin.

To form the final temperature-sensing yarns, the carrier fibres, interconnects and the micro-pod were surrounded with six (167 dtex/48 filament) polyester yarns (packing fibres in Figure 1) and a circular warp-knitted structure (knit braid; RIUS MC braiding machine, RIUS, Barcelona, Spain), which was produced using six additional polyester yarns (167 dtex/48 filaments).

The electronic temperature-sensing yarns used for the calibration experiments and for the prototype sock had 0.87 mm-diameter resin micro-pods.

The temperature-sensing yarns broke at 60.0 N, with 11% elongation, as tested using a tensile testing machine (Z2.5, Zwick/Roell, Ulm, Germany) in accordance with ASTM D 2256-02 (ASTM International, West Conshohocken, USA). This is higher than the breaking point of standard fibres, such as polyester, and therefore made the yarns suitable for undergoing the knitting process. The fibres covering the electronic components of the yarn broke during testing before the electronic elements were damaged.

### 2.2. Supporting Hardware for Temperature Measurements

For all experiments (unless otherwise stated) thermistors were connected to a dedicated analogue-to-digital converter (ADC) via a voltage divider circuit. Experiments investigating response time used a Teensey v3.2 microcontroller (PJRC, Sherwood, OR, USA) to preform analogue-to-digital conversion with voltage measurements taken using 13-bits at a sampling rate of 1.3 kHz. The voltage divider used 9.8 kΩ resistors. The Arduino v1.6.12 (Arduino LLC, Boston, MA, USA) and Teensyduino v1.35 (PJRC, Sherwood, OR, USA) software were used to record temperature measurements. This system closely represented the recording hardware that could be employed in a prototype garment, given its small size.

Data presented for the prototype garments used a PICO-TC08 (Pico Technology, Cambridge, UK) as an ADC and an NI USB 6008 DAQ data acquisition device (National Instruments™, Austin, TX, USA).

The values of the resistors used in the potential divider circuits were determined with a digital multi-meter (Agilent 34410A 6½ digit, Agilent Technologies, Santa Clara, CA, USA) to a precision of 0.01%.

### 2.3. Response of Electronic Temperature-Sensing Yarns

#### 2.3.1. Step Response Time Theory

The step response time is a vital parameter for temperature sensors as it determines the temporal resolution of the sensor. Since the thermistor in the electronic temperature-sensing yarn was not in direct contact with the surface being measured, the heat had to be transferred through the polymer resin and the polyester filaments, which provided a thermal resistance, restricting the heat flow. The step response time of a measuring system is the time taken by the measuring system to reach a final steady value, once it is subjected to an abrupt change between two specified, constant quantities [23].

In the simplest case the response to a temperature change will follow Newton’s law of heating (or Newton’s law of cooling) which describes the temperature change of an object as a function of time when there is a temperature differential between the object and its surroundings [25]. Ignoring radiative heat transfer, this gives the following relationship:

(1) $$T\left(t\right)=T_{s}-\left(T_{s}-T_{0}\right)e^{-\frac{t}{r}}$$

where *T*(*t*) is the temperature at a given time *t*; *T_s_* is the temperature of the surroundings; *T*_0_ is the initial temperature of the object; and *r* is the characteristic thermal time constant.

The sensor step response time is the time taken for the sensor to reach a new equilibrium condition after a change. Due to the exponential nature of this relationship, equilibrium will be achieved after 5 × *r*.

#### 2.3.2. Response Time Experiments

To understand the potential limitations of the technology, two heat-transfer conditions were investigated—the thermistors placed into an oil bath, and the thermistors placed directly onto a precision hotplate. For both conditions, a precision electronic hot plate (Model 1000-1, Electronic Micro Systems Ltd., Salisbury, UK) was used. When the oil bath was placed on top of the hotplate, the plate was set to 45.0 °C which resulted in an oil temperature of ~38 °C (monitored with a K-type thermocouple and reader; Six Channel Handheld Temperature Data Logger RDXL6SD, Omega Engineering, Inc., Manchester, UK) with fluctuations not exceeding ±1 °C, and normally much less within single datasets. When samples were placed directly on the plate, the hotplate was set to 38.0 °C. While the laboratory was not temperature-controlled, room temperatures of 25 ± 1 °C were recorded during the experiment(s). These temperatures (38 and 25 °C) were chosen to represent skin temperature and an ambient environment respectively. Healthy skin has an external temperature of 34 °C, so a value greater than this was chosen in order to test the widest range of temperatures that would be likely to be encountered in normal use. For the oil bath, 80 mL of pure sunflower oil (Tesco PLC, Welwyn Garden City, UK) in a glass beaker was heated until the temperature had stabilized. The oil was not stirred to limit convection currents.

All test samples (thermistors, encapsulated thermistors, electronic temperature-sensing yarns) were initially held at room temperature before placement into the oil bath or onto the hotplate. For the latter experiment, double-sided tape was used to ensure good contact between the plate and sample; the tape was always attached to the hotplate and therefore held the same temperature as the plate itself. Samples were then heated for 6 min, ensuring that full equilibrium had been reached, before being removed. The recording of the temperature then continued for an additional 9 min. In this manner the data for both heating and cooling were obtained.

Data fittings were then carried out using IGOR Pro (Version 7.0.2.2; Wavematrics, Tigard, OR, USA) to acquire the characteristic time constant for heat and cooling. In most cases, four repeat measurements were taken with the standard deviation of these readings showing the level of error.

Additionally, the validity of the absolute temperature readings were checked by averaging 285 data points from the stable region once an equilibrium had been reached.

### 2.4. Calibration of Sensor Yarn

A rigorous calibration was conducted on the electronic temperature-sensing yarns to validate the calibration data provided by the thermistor manufacturer and to ensure that the presence of the yarn encapsulation did not affect the response of the encapsulated sensor. The calibration procedure consisted of heating five electronic temperature-sensing yarns in the oil bath (as described in Section 2.3.2), along with an NTC Thermistor (USP11493, US Sensor Corp., Orange, CA, USA, accurate to ±0.05 °C). The entire ensemble was contained within a thermally insulating polystyrene box and temperatures were held for 40 min to ensure that convection effects were minimised. For these experiments, the thermistor resistances were obtained using a high-accuracy digital multi-meter (Agilent 34410A).

**Figure 2:**
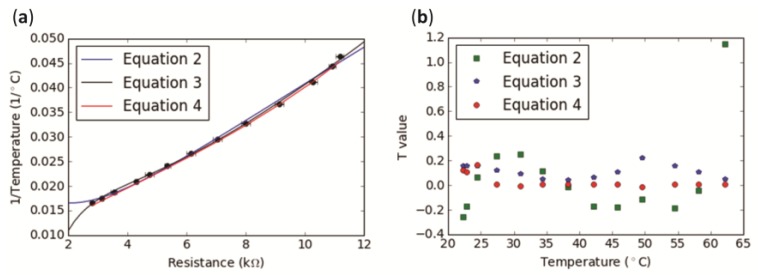
Calibration showing readings from five electronic temperature-sensing yarns (ETS yarns). (**a**) The averaged values of the resistances of the five electronic temperature-sensing yarns have been plotted (black markers) against the reciprocal of the temperature. Two polynomial fittings were applied as well as the manufacturer’s equation to calculate the reciprocal of temperature; (**b**) Residuals for each of the Equations (2)–(4).

Figure 2, showing the calibration data, was produced using Python v2.7 (Python Software Foundation, Wilmington, DE, USA) using the SciPy [26] and Matplotlib [27] packages.

### 2.5. Prototype Temperature-Sensing Sock

Electronic temperature-sensing yarns were produced and integrated into a prototype temperature-sensing sock. The prototype sock was produced using five electronic temperature-sensing yarns positioned at different points on the sole of the sock for remote temperature monitoring. It was of most interest to measure the temperature on the big toe, heel, and metatarsal heads, which informed the placement of the sensors. Given the size of the metatarsal heads, three sensors were placed there.

Thermistors were positioned correctly by inserting the electronic yarns into 2.0 mm diameter channels within a seamless knitted sock. The sock with the channels was produced using a computerized flatbed knitting machine (Model SWG 091N3, 15NPI, Shima Seiki, Sakata Wakayama, Japan).

In order for the sock to provide remote temperature measurements, the sensor yarns were connected to a microcontroller (Arduino Pro Mini, Arduino, Scarmagno, Italy) and Bluetooth module (BlueSmirf, Sparkfun Electronics, Boulder, CO, USA). LabVIEW (2014 SP1, National Instruments™, Austin, TX, USA) was used to program and create a graphical user interface.

## 3. Results and Discussion

### 3.1. Electronic Temperature-Sensing Yarn Calibration

The resistance values from five electronic temperature-sensing yarns with embedded thermistors were averaged and plotted against the reciprocal of the temperature (in a range spanning 22.25 to 62.15 °C, going beyond the extreme limits of the temperature range of interest). This data first had a polynomial approximation fitting applied to convert the temperature-sensing yarn’s resistance into temperature [28] (Figure 2a), with the calibration fitting given by Equation (2). Temperatures for the calibration were recorded using the US Sensor Corp. Thermistor, which was more accurate (to ±0.05 °C) than the thermistors in the temperature-sensing yarn.

(2) $$\frac{1}{T}=0.017598-0.003687[\ln\left(R\right)]+0.002545{\left[\ln\left(R\right)\right]}^{3}$$

where *T* is the temperature (in °C) and *R* is the resistance (in kΩ).

Recent literature has shown a more accurate calibration fitting. The data used previously were therefore fitted with the new polynomial approximation, once again converting the thermistor resistance into the reciprocal of temperature [29] (Figure 2a), with the second calibration fitting given in Equation (3).

(3) $$\frac{1}{T}=0.040576+0.041704\ln\left(\frac{R}{R_{0}}\right)+0.030514[\ln\left(\frac{R}{R_{0}}\right){]}^{2}+0.009980[\ln\left(\frac{R}{R_{0}}\right){]}^{3}$$

where *T* is the temperature (in °C); *R*_0_ is the resistance at 25 °C (in kΩ); and *R* is the resistance (in kΩ).

It was also important to clarify whether the equation given by the thermistor manufacturer could be used to provide accurate temperatures. Therefore, average resistance values (*Rt*) from the electronic temperature-sensing yarns were used to calculate the reciprocal of the temperature (1/*T*) using Equation (4).

(4) $$\frac{1}{T}=\left(\frac{\ln\left(\frac{Rt}{R_{0}}\right)}{B}\right)+\left(\frac{1}{T_{0}}\right)$$

where *R*_0_ is 10 kΩ ± 1% resistance at *T*_0_, which is 298.15 K (25 °C); and *B* is the material constant (3395 for the temperature range of 20–65 °C, provided by the manufacturer). The temperature (*T*) obtained was in Kelvin and was converted to °C. Thereafter the reciprocal of this temperature was plotted as shown in Figure 2a.

Residuals for each of the equations were calculated in order to identify which of the fitting equations (Equations (2), (3), or (4)) would provide the most accurate temperature measurements compared to the US Sensor Corp. thermistor. The residuals give the significant difference between the population mean and a hypothesized value. The average of the residuals over the temperature range of interest gave 0.03554 for the manufacturer’s equation, 0.2274 for Equation (2) and 0.1157 for Equation (3).

Equation (2) gave the largest deviation between the temperature obtained from the electronic temperature-sensing yarn resistances and that of the calibrated thermistor (Figure 2). Equation (4) provided the values with the closest agreement. Residuals as a function of temperature are shown in Figure 2b.

Figure 2a shows that the all of the fittings were in good agreement with the data. Equation (4) fitted the data well within the error bars. It can be observed from Figure 2b that the Equation (4) provided the lowest residual values and was therefore the most accurate fitting of those explored. Hence, for this paper, the manufacturer’s equation was used to calculate the temperature from the thermistor.

At this point the true accuracy of the electronic temperature-sensing yarn was also assessed. It was possible that the direct conversion of the thermistor resistance tolerance to a temperature error may have been underestimated given that the thermistor’s encapsulation process could have introduced additional sources of error. Equation (4) was used to calculate the temperatures based on the resistance values collected from the temperature-sensing yarns. These calculated values were then compared to the temperature given by the US Sensor Corp. thermistor.

It should be noted that of the five yarns, one yarn (Yarn 3) had values that differed from the calibration thermistor significantly more than the others. In the 22.25 to 62.15 °C temperature range 89% of the yarn measurements were within 1.0 °C of the calibration thermistor values, and 63% were within the desired 0.5 °C when the Yarn 3 data were included: Removing Yarn 3 gave 98% and 73% for ±1.0 and ±0.5 °C, respectively. This was deemed acceptable for this study and future work will strive to improve measurement accuracy, as a ±1.0 °C error in the temperature reading from two yarns would be enough to give a false result for ulcer formation.

There were a number of potential causes for the differences seen between the electronic temperature-sensing yarns and the calibration thermistor. The most likely reason was due to the manufacturing tolerances of the electronic temperature-sensing yarns. Firstly, the thermistors are known to have a tolerance, and this is reported by the manufacturer as ±1%. Minor differences in the encapsulation and soldering processes could further add to this error. Age was also a consideration, as thermistor values are known to shift slightly with use and prolonged exposure to higher temperatures [28]. Ultimately, it would be desirable to use a thermistor for the yarns that had a lower resistance tolerance as this would reduce the error in temperature measurements.

The manufacturer’s equation (Equation (4)) for converting resistance into temperature for the thermistors was seen to provide accurate and representative values for the temperature-sensing yarns. While other data fitting methods were explored, Equation (4) gave the best agreement with the highly accurate US Sensor Corp. thermistor measurements. As a result, Equation 4 was used to convert resistance to temperature throughout this work.

### 3.2. Response Time of Encapsulated Thermistor and Stability of Readings

#### 3.2.1. Step Response Time

**Figure 3:**
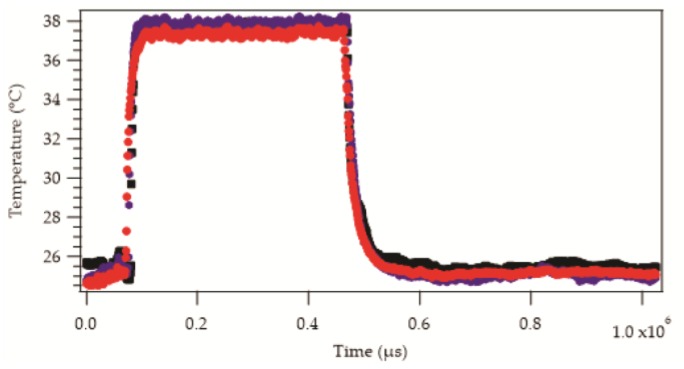
Sample data set showing the temperature response of three different samples when placed onto the hotplate (38 °C) and then removed to room temperature (25 °C). The temperature reading for all of the samples increased to an equilibrium value exponentially. Similarly, cooling shows an exponential relationship. The diameters of the resin micro-pods are as follows: 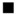 = 0.70 mm, 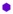 = 1.00 mm, 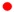 = 1.90 mm. Markers appear as lines in the figure due to the density of data.

As expected, the placement of the samples into the oil bath or onto the hotplate resulted in a rapid increase in the recorded temperature. Removing the samples from the source of heat brought about a drop in the recorded temperature. An example of the raw temperature data as a function of time from six samples is shown in Figure 3.

**Figure 4:**
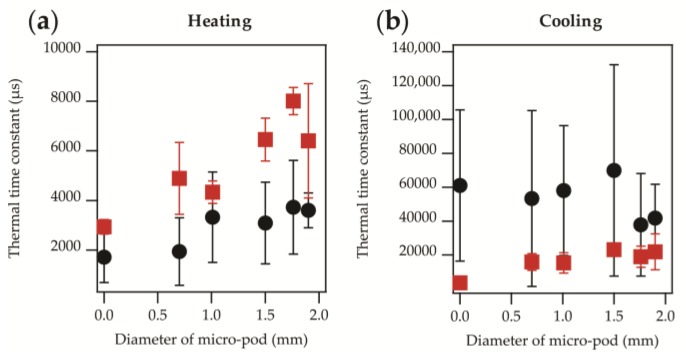
Thermal time constants as a function of the micro-pod diameter. Data presented shows experiments where an oil bath was used (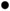) or the samples were attached directly to a hotplate (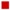). (**a**) Heating time constant; (**b**) Cooling time constant. In both cases the effect of the micro-pod on the time constants were negligible within the experimental error.

The absolute temperatures appeared to be similar in most cases but not identical. The increase and decrease in temperature appeared to be exponential in nature, as would be expected from Newton’s law of heating or cooling (see Equation (1)), with some variation in the rates. This was confirmed by applying data fittings, as described in Section 2.3.1, to obtain heating and cooling time constants. These are displayed for heating and cooling in Figure 4a,b.

It was clear that heating of the samples gave a shorter time constant than cooling, with a difference of over a factor of thirty between the constants in some cases. It is known that thermal time constants can be substantially different for the heating or cooling of thermistors [30].

It was of interest to note that for both heating and cooling there was an offset in the time constant depending on whether the oil bath had been used or the sample had been attached to the plate directly. When using the oil bath the samples took less time to reach equilibrium when heated, and longer to cool compared to when the samples were attached to the plate directly. The greater cooling time constant, observed when samples were removed from the oil bath, was due to the layer of oil that clung to the surface of the samples. This heated oil layer acted as an additional thermally resistive barrier that slowed the cooling process. It is important to note that the differences between the results were minimal within the experimental error (as shown by the error bars in Figure 4). A shorter time constant for heating was likely due to the entire yarn being submerged in the oil, therefore heating would occur on all sides of the yarn and not just on one side (i.e., the side of the thermistor which is in direct contact with the heating surface) as was the case with the hotplate.

Ultimately, the presented data gave thermal time constants between 1716–8012 μs when heating, and 3595–69,898 μs when cooling. The corresponding step response times were 8578–40,061 μs (0.01–0.04 s) for heating and 17,976–349,490 μs (0.02–0.35 s) for cooling. A maximum step response time of 0.35 s would be negligible for most skin temperature monitoring applications—temperature changes due to ulcer formation or wound infection would occur over a period of the order of hours.

Preliminary experiments were conducted with a resin type with superior thermal transfer characteristics (Dymax Multi-Cure^®^ 9-20801, Dymax Corporation, Torrington, CT, USA; thermal conductivity: 0.9 Wm^−1^K^−1^), which provided shorter time constants compared to the Dymax 9001-E-V-3.5, as would be expected. Given that the thermal time constants obtained when the Dymax 9001-E-V-3.5 was used were sufficiently short for this application, full characterization using the Dymax 9-20801 was not conducted.

**Figure 5:**
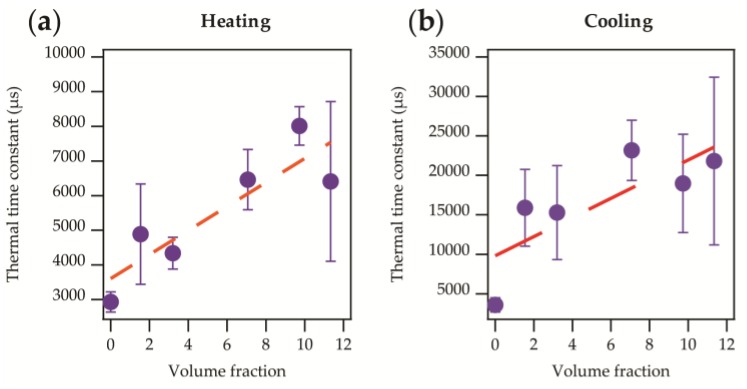
Thermal time constant against the volume fraction of resin surrounding the thermistor compared to the thermistor’s volume. Data presented shows when samples were directly attached to the hotplate (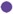; previously shown in Figure 4a). (**a**) Heating thermal time constant. A direct linear relationship (r^2^ = 0.776) is observed as shown in Equation (5); (**b**) Cooling thermal time constant. The linear relationship (r^2^ = 0.629) observed is shown in Equation (6).

A linear relationship between the thermal time constant and micro-pod diameter was clearly apparent when samples were attached to the hotplate directly (as shown in Figure 4). This was due to the greater volume of resin around the chip causing a restriction to heat flow, and therefore increasing the thermal time constant as a function of the micro-pod size. A relationship was less clear when the oil bath was used due to a greater level of variation in the results, as shown by the error bars. To better understand this effect, the volume fraction of resin surrounding the thermistor was calculated and compared to the volume of the thermistor itself. The volume fraction was then plotted against the thermal time constant as shown in Figure 5.

In Figure 5a there is a clear linear relationship between the volume fraction and thermal time constant (Equation (5)), with the thermal time constant increasing with a greater volume fraction.

(5) Thermal time constant=3606.1±643.0−346.7 ±93.1× Volume fraction 

Figure 5b gives a similar linear relationship (Equation (6)).

(6) Thermal time constant=9806.6±3210.0−1213.6 ±466.0× Volume fraction 

The intercept is the intrinsic thermal time constant of the thermistor and could be expected to be different during either heating or cooling [30]. It was important to note that neither fitting that was presented represented the un-encapsulated thermistor well. The gradients also differed notably, by a factor of 3.5. Heating and cooling should have occurred at the same rate if conditions were identical, hence if the relationship between volume fraction and time constant was purely due to a restriction of heat flow, the same effect should be observed for both heating and cooling. The difference observed may have been due to heat transfer mechanisms occurring as the samples cooled (principally convective) as opposed to heated (principally conductive).

For a practical application of the types described, where the garment with temperature-sensing yarns would have close contact with the skin, the latter was of most interest as conductive heat transfer would be dominant. This was a useful general relationship to understand, as future iterations of this technology may employ other thermistor types (that are physically larger or smaller). This general solution can be applied if a new temperature sensor type is employed. Similarly, this may prove useful for engineering the encapsulation for devices that heat up, such as LEDs.

#### 3.2.2. Step Response Time for Electronic Temperature-Sensing Yarns with a Fibre Sheath

Once the thermally resistive effects of the resin micro-pod encapsulation were understood, two sets of experiments were conducted using electronic temperature-sensing yarns to determine how the fibre layers affected the step response time. Two electronic temperature-sensing yarn samples were attached directly to the hotplate (the oil bath was not used). Two conditions were investigated, using a temperature-sensing yarn of approximately 1.5 mm diameter, and using a temperature-sensing yarn encased in a secondary knitted sleeve (approximately 4 mm diameter), to better represent a system where a temperature-sensing yarn had been embedded within a textile. In both cases the thermal time constants for heating were longer than those when only a resin micro-pod was used as encapsulation. The heating thermal time constants were 8210 ± 1444 μs and 34,175 ± 13,263 μs, and cooling time constants 19,440 ± 7339 μs and 37,425 ± 2246 μs for the electronic temperature-sensing yarn with the additional sleeve and the electronic temperature-sensing yarn, respectively. This corresponded to step response times for heat of 41,050 and 170,875 μs (0.04 and 0.17 s) and cooling of 97,200 and 187,125 μs (0.01 and 0.19 s) for the electronic temperature-sensing yarn with the additional sleeve and the electronic temperature-sensing yarn, respectively. It was interesting to note that the example with the additional knitted sleeve, and therefore more fibres between the thermistor and hotplate, gave shorter time constants.

The shorter time constant may relate to the thermal properties of the knitted structure due to air being trapped within the knitted loops. This potentially trapped warm air may create a micro-climate around the yarn, limiting heat loss through the top of the yarn (which was not in contact with the hotplate), and allowing the embedded thermistor to heat up more rapidly.

These results support the earlier assertion that the porous knitted structure itself has a limited effect on the heat flow to the thermistor. This was reinforced by the fact that the cooling time constants were not substantially different to those shown in Figure 4.

The above time constants gave a maximum step response time of 0.17 ± 0.07 s for heating, or 0.19 ± 0.01 s for cooling.

#### 3.2.3. Stability and Accuracy of Temperature Readings

**Figure 6:**
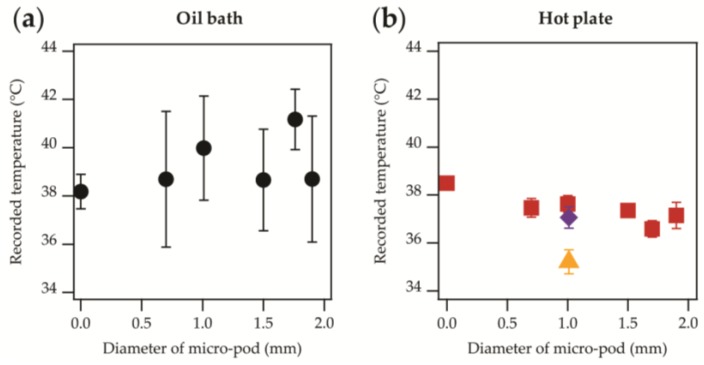
Averaged temperature values once an equilibrium was reached for samples with micro-pod diameters from 0 mm (unencapsulated) to 1.90 mm. (**a**) Data presented shows experiments where an oil bath was used (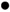); (**b**) The samples were attached directly to a hotplate (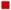). Data from samples that have included covering fibres; temperature-sensing yarn (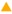), temperature-sensing yarn with an additional fibrous sheath (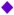).

Figure 6 shows the recorded temperature of samples with different micro-pod diameters, once an equilibrium condition had been reached.

When the oil bath was used (~38 °C), averaged temperatures spanned between 38.184–41.169 °C, meaning that the temperature readings were in agreement within the measurement error (with one exception: 1.76 mm diameter encapsulation). The large experimental errors were likely to be due to convection effects in the oil caused by the insertion of the yarns into the bath (the heating step). The results of greater interest were those when the samples were placed directly onto the hotplate. This provided a 38 °C temperature to one side of the thermistor and exposure to the ambient temperature (25 °C) on the other. This closely represents the conditions that the system would likely experience in normal operation (in contact with skin on one side and air on the other). Here, the variation in the results was more restricted, with standard deviations ranging between 0.02 and 0.545 °C. The range of the averaged temperatures (for four measurements) covered 36.592–38.497 °C, or covered 36.592–37.631 °C when the results from the un-encapsulated package die were excluded. This gave a higher temperature reading (Figure 6). It was not clear whether the lack of encapsulation meant that this higher temperature reading of 38.5 °C was achieved, or whether this discrepancy of up to 1.9 °C was within the manufacturing tolerance of the thermistors. Additional experiments (not presented) have suggested that a larger temperature difference between the hotplate and ambient temperature resulted in a greater change in the recorded temperature when encapsulation was used. This would be due to the greater temperature gradient and therefore the energy losses in the system would mean that thicker resin micro-pods would never reach the same temperature as the hotplate.

Stability data from the two samples where covering fibres have been included have also been presented (Figure 6). The data from the electronic temperature-sensing yarn with an additional fibre sheath (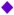) is in agreement with the other data. It is possible that the yarn with the additional fibrous sheath gave higher recorded temperatures than when only one sheath was used (i.e., normal temperature-sensing yarn; 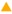) due to warm air becoming trapped within the loops of the knitted structure, and small air-gaps between the sheath and temperature-sensing yarn. As discussed earlier, this may create a microclimate around the temperature-sensing yarn, minimizing heat losses to the environment. It is important to highlight that within a textile the temperature-sensing yarn would be surrounded by an additional fibrous layer, making the temperature-sensing yarn within an additional fibre sheath a more representative model of the yarns in their final intended use.

For wound care or diabetic ulcer formation a temperature difference is desired, and not an absolute temperature. Therefore, any ambiguity about the absolute temperature measurements is not relevant for these applications as a relative measurement is suitable for predicting foot ulceration or wound infection. In potential future applications where the yarns might experience a greater temperature differential an absolute temperature reading could be required. Further work may be necessary to better characterize this effect.

### 3.3. Prototype Temperature-Sensing Sock

**Figure 7:**
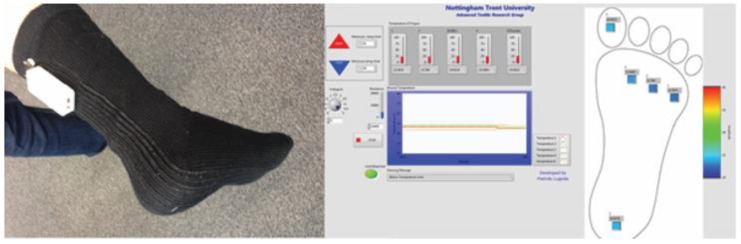
Prototype sock made with five electronic temperature-sensing yarns. (**Left**) Photograph of the prototype; (**Right**) Computer interface using LabVIEW. The interface shows temperature readings for five areas on the prototype garment using both numbers (not visible here) and a colour change.

A temperature-sensing sock capable of remotely monitoring skin temperature was produced to show the utility of a network of electronic temperature-sensing yarns in a garment. Electronic temperature-sensing yarns were incorporated into the knitted, prototype sock. The sock was tested on one of the investigators as shown in Figure 7 (left). The sensor network provided temperature measurements of 29.4 °C, 29.2 °C, 29.9 °C, 29.6 °C, 29.6 °C from five points in contact with the sole of a foot. While these absolute temperatures were lower than body temperature they were within one standard deviation of mean measured foot temperatures presented in the literature (30.6 ± 2.6 °C) [31]. The relative measured temperatures differed by a maximum of 0.7 °C (ultimately, the relative measurement is of interest for foot ulcer formation).

## 4. Conclusions

Thermistors were encapsulated within yarns, and subsequently within textiles, to create a novel skin temperature monitoring solution. The electronic temperature sensor yarns were characterized to establish their suitability for monitoring diabetic foot ulcer formation and wound infection, which informed the temperature range and conditions explored within this work.

The formation of a polymer micro-pod to protect the thermistor is a critical step in chip encapsulation and the effects of this process have been fully investigated for these temperature monitoring applications. It was observed that very short step response times of 0.01–0.04 s when heating or 0.02–0.35 s when cooling were achievable. When the encapsulated thermistors were placed within electronic temperature-sensing yarns a maximum step-response time of 0.17 ± 0.07 s for heating, or 0.19 ± 0.01 s for cooling was calculated. This was well within the temporal resolution necessary for a sensor of this type, where temperature changes would occur over the course of hours.

In the temperature range of interest, the inclusion of the micro-pod showed a functional relationship between the response time (a function of the thermal time constant) and micro-pod diameter. This relationship has been explored to provide a more general solution for the way in which encapsulation may affect temperature readings for other thermistor sizes or the heat transfer through a micro-pod in other applications (for example an encapsulated device such as an LED heating up).

The accuracy of the electronic temperature-sensing yarns was determined to be within ±0.5 °C for 63% of measurements as shown when the calibration of the yarns was performed. This was further supported by the data shown in Figure 6b. This level of accuracy (±0.5 °C for 63% of measurements) may not be sufficient for the application of detecting diabetic foot ulcer formation and if it is found to be the case then future work will need to investigate the use of more accurate temperature-sensing elements to improve this

The creation of a remote, representative, prototype temperature-difference monitoring device (a sock) for skin measurements opens up a number of exciting possibilities for both clinical and pre-clinical studies. In future studies, a system-level evaluation of the presented sock will be conducted to ready the technology for use in potential pre-clinical studies.

Future work will also exploit this innovation to address the apparent absence in the literature of guidance on the temperature variation during wound infection.

In conclusion, the novel electronic temperature-sensing yarn presented here has been shown to be well-suited for making long-term measurements of temperature when incorporated into a garment. The yarn was used to create an innovative skin-temperature-difference monitoring device capable of providing remote, continuous temperature measurements at given points on the skin. The miniature size of the temperature sensors makes them invisible to the end user and is well-suited for wearable applications.
